# Supplementary material for: The Construction and Exploration of a Comprehensive MicroRNA Centered Regulatory Network in Foxtail Millet (Setaria italica L.)
Source: Front Plant Sci. 2022 May 6;13:848474. doi: 10.3389/fpls.2022.848474 (PMC9121102; doi:10.3389/fpls.2022.848474)
Supplement: Supplementary file 3 [file Data_Sheet_3.zip › Sit-miR2275b_Seita.1G097800.1_5746_TPlot.pdf]

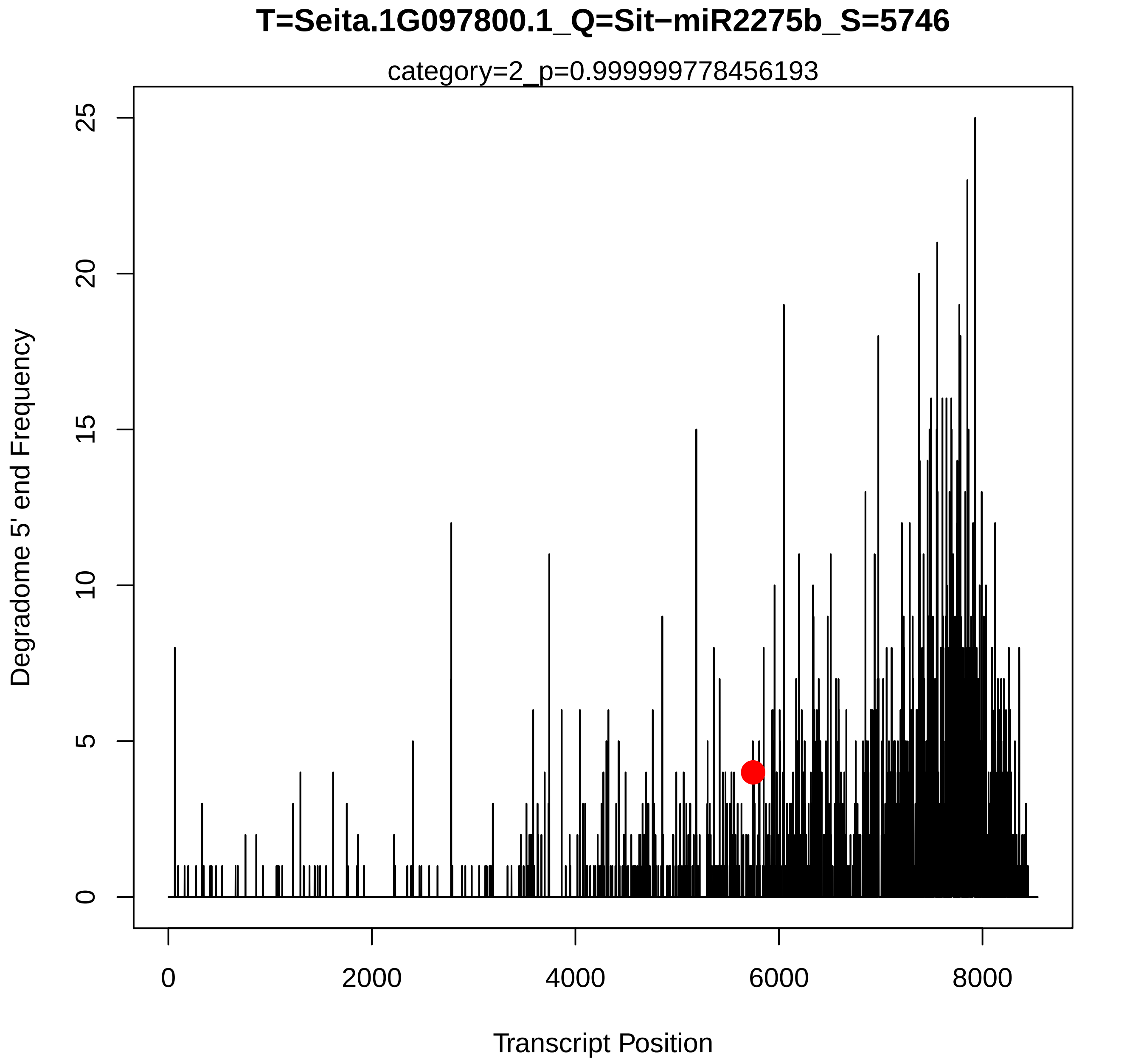

T=Seita.1G097800.1_Q=Sit−miR2275b_S=5746
category=2_p=0.999999778456193
Degradome 5' end Frequency
0
5
10
15
20
25
0
2000
4000
6000
8000
Transcript Position
